# Isolation of the Endophytic Fungus *Aspergillus terreus* from a Halophyte (*Tetraena qatarensis*) and Assessment of Its Potential in Tomato Seedling Protection

**DOI:** 10.3390/plants13162218

**Published:** 2024-08-10

**Authors:** Fedae Alhaddad, Talaat Ahmed, Samir Jaoua, Mohammad A. Al-Ghouti, Roda Al-Thani, Mohammed Abu-Dieyeh

**Affiliations:** 1 Biological Science Program, Department of Biological and Environmental Sciences, College of Arts and Sciences, Qatar University, Doha P.O. Box 2713, Qatar; fa1108048@student.qu.edu.qa (F.A.); ralthani@qu.edu.qa (R.A.-T.); 2 Environmental Science Center, Research and Graduate Studies, Qatar University, Doha P.O. Box 2713, Qatar; t.alfattah@qu.edu.qa; 3 Environmental Science Program, Department of Biological and Environmental Sciences, College of Arts and Sciences, Qatar University, Doha P.O. Box 2713, Qatar; samirjaoua@qu.edu.qa (S.J.); mohammad.alghouti@qu.edu.qa (M.A.A.-G.)

**Keywords:** endophyte, biocontrol, halophyte, antagonism, growth promotor

## Abstract

Living in diverse environmentally harsh conditions, the plant exhibits a unique survival mechanism. As a result, the endophytes residing within the plant produce specific compounds that promote the plant’s growth and defend it against pathogens. Plants and algae symbiotically harbor endophytes, i.e., microbes and microorganisms living within them. The objective of this study is to isolate endophytic fungi, specifically strains of *Aspergillus terreus,* from the leaves of the salt-tolerant plant *Tetraena qatarensis* and to explore the salt tolerance, antagonistic activity, and growth promotion properties. Strain C *A. terreus* (ON117337.1) was screened for salt tolerance and antagonistic effects. Regarding salt tolerance, the isolate demonstrated the ability to thrive in a concentration of up to 10% NaCl. *A. terreus* showed inhibitory activity against four fungal phytopathogens, namely *Fusarium oxysporum, Alternaria alternata*, *Colletotrichum gloeosporioides,* and *Botrytis cinerea.* The GC-MS investigation of the fungal (strain C *Aspergillus terreus*) extract showed the presence of about 66 compounds (secondary metabolites). Secondary metabolites (SMs) are produced, like Hexadecanoic acid, which aids in controlling phytopathogens. Also produced is lovastatin, which is used to treat hypercholesterolemia. Strain C, which showed salinity tolerance and the highest inhibitory activity, was further analyzed for its effect on tomato seed germination under pathogen stress from *Fusarium oxysporum*. The greenhouse experiment indicated that the fungi increased the length of tomato seedlings and the plant biomass. Therefore, the selected endophytes derived from *Tetraena qatarensis* were scrutinized for their potential as biocontrol agents, aiming to thwart fungal pathogens and stimulate plant growth. The in vitro and in vivo assessments of strain C (*Aspergillus terreus*) against *Fusarium oxysporum* in this investigation indicate the promising role of endophytes as effective biological control agents. Investigating novel bio-products offers a sustainable approach to agriculture, gradually reducing dependence on chemical fungicides.

## 1. Introduction

In the year 2050, the global population is projected to experience a substantial increase, reaching an estimated 9.7 billion. This surge in population will inevitably lead to an escalated demand for food resources [1,2]. Recognizing this challenge, the Food and Agriculture Organization (FAO) of the United Nations underscores the necessity for a minimum 50% augmentation in average agricultural food production by the year 2050 [2]. Consequently, it becomes crucial to formulate a comprehensive strategy aimed at mitigating pre- and post-harvest crop losses, with the overarching goal of enhancing overall crop production to align with the escalating needs of the global population [1]. A growing awareness of agricultural chemicals’ potential harm has emerged in recent years. As a result, organic farming practices have become increasingly popular, as it offers a range of benefits, such as reducing harmful chemicals in food, improving crop nutrition, promoting soil health, and maintaining a natural balance of microorganisms in the ecosystem. To mitigate the negative impact of chemical-based agriculture, plant-growth-promoting microbes, or biofertilizers, are now commonly used. Seeds, plants, and soil can be inoculated with biofertilizers, which are live microbial inoculants. Biofertilizers also play an essential role in promoting sustainable growth for the plants. These microbes colonize both the internal and external environments of the plant, aiding in nutrient solubilization and the production of essential metabolites to support plant growth over an extended period [3]. 

Endophytes are located within the tissues of plants and have direct, potent interactions with their hosts [4]. A solitary plant can harbor a diverse array of fungal endophytes [5]. The varied qualities displayed by these fungi, which promote plant growth, contribute positively to plants by providing both biotic and abiotic stress tolerance. This enhances the overall health of plants, particularly in challenging conditions [6]. Endophytes may enhance host protection by boosting the activation of the host’s inherent defense mechanisms and supplying supplementary defense resources beyond those of the host itself [7]. By releasing various ranges of secondary metabolites (SMs), these fungi activate the plant’s defense mechanism against plant pathogens [8]. Numerous fungal endophytes found in cotton have been identified for producing biologically active compounds aimed at preventing plant diseases [9]. The fungi are a valued reservoir of bioactive compounds [10,11,12]. They produce important secondary metabolites like gibberellins and compounds with immunosuppressive properties, as well as anticancer, antidiabetic, antifungal, and antibacterial characteristics [13,14,15]. There is evidence suggesting that specific endophytes, producing unique phytochemicals initially linked to their host, may be associated with a genetic partnership that has evolved gradually between the endophyte and the host plant [16,17]. 

Studies have suggested that endophytic fungi (EF) found in association with halophytes offer beneficial and eco-friendly additives for improving the growth of plants in saline soils [18]. This has prompted growing attention towards investigating and discovering the potential of endophytic resources associated with halophytes [19,20]. 

*Tetraena qatarensis* (Hadidi) is a halophytic plant belonging to the Zygophyllaceae family. Indigenous to the Arabian Peninsula and Persia, *T. qatarensis* demonstrates resilience to salinity and drought, classifying it as both a halophyte and xerophyte [21,22]. Lately, there has been a rising interest in researching *T. qatarensis* owing to its ecological importance, distinctive physiological adaptations, and potential applications in various fields. Consequently, the current study is dedicated to isolating the fungal endophyte *Aspergillus terreus* found in association with the surface-sterilized leaves of the halophyte *T. qatarensis*.

The fungi *Aspergillus terreus* are found naturally in the environment [23]. In the literature, the genus *Aspergillus* in general produces a wealth of advantageous bioactive metabolites with antibacterial and antifungal properties [24]. Research has demonstrated the antifungal efficacy of a compound derived from *A. terreus*, effectively inhibiting plant pathogenic fungi such as *Rhizoctonia solani* [25]. Additionally, the bioactive metabolites of *A. terreus* were found to impact the control of *Pythium ultimum* [26]. 

This study has the following objectives. The primary goal is to isolate fungal endophytes (*Aspergillus terreus*) from halophytic plants and assess their salinity tolerance, aiming to identify a distinct isolate thriving in these specialized ecological environments. The secondary objective is to explore the antagonistic properties of the isolated endophyte against fungal pathogens. Understanding the interactions and potential control mechanisms these microbes have over fungal pathogens is crucial for investigating their role in biocontrol strategies. The third objective is to evaluate the specific impact of the *A. terreus* isolate C on *Fusarium oxysporum*, a notable fungal pathogen. Furthermore, preliminary observations suggest the growth-promoting effects of this highly efficient isolate on tomato plants under the influence of infection by the fungal pathogen *F. oxysporum*. 

## 2. Results

### 2.1. Identification of Fungal Isolate 

**Figure 1:**
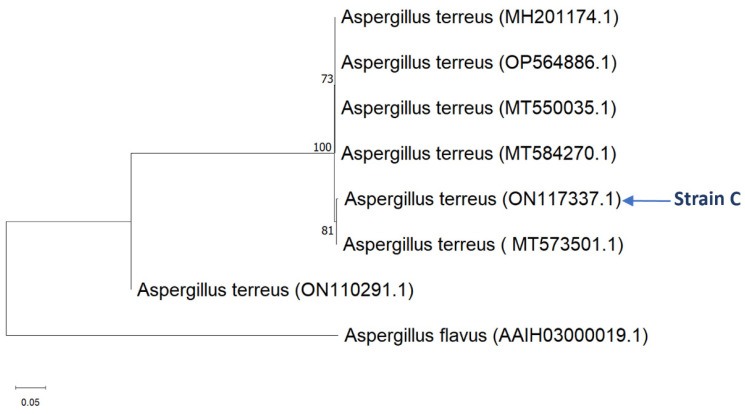
Molecular phylogenetic tree analysis for *Aspergillus terreus* by maximum likelihood method with the value of (1000) bootstrap comparisons. Evolutionary analyses were conducted by [MEGA 11].

The fungal strain was isolated from the inner tissues of leaves of the halophyte *Tetraena qatarensis*. Total genomic DNA from the isolated strain was extracted and analyzed using the ribosomal RNA (ITS) region. Fragments ranging from 550 to 610 bps were amplified using the universal primer pair (ITS1 and ITS4). The PCR products were sequenced and compared to the sequences in the NCBI GenBank database. Obtained sequences were deposited in GenBank under the accession number (ON117337.1). Results revealed a 99% similarity with *Aspergillus terreus*. The phylogenetic tree of the maximum likelihood method was constructed using (MEGA 11) software www.megasoftware.net, as shown in Figure 1.

### 2.2. Salt Tolerance Evaluation 

**Figure 2:**
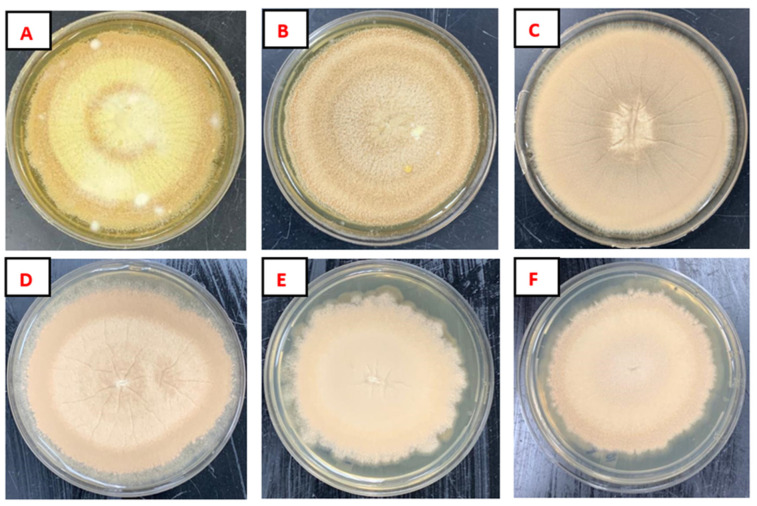
Mycelium growth of strain C (*A. terreus*) on PDA plates mixed with different salinity gradients at 25 °C. (**A**) 0% NaCl, (**B**) 2% NaCl, (**C**) 4% NaCl, (**D**) 6% NaCl, (**E**) 8% NaCl, and (**F**) 10% NaCl.

 The evaluation of salinity tolerance of *Aspergillus terreus* isolate was performed in PDA media inoculated with different NaCl concentrations (0%, 2%, 4%, 6%, 8%, and 10% of NaCl). The results revealed that *A. terreus* has the ability to grow in a concentration of up to 10% NaCl, as shown in Figure 2. The mycelium growth diameters were measured and are recorded in Table 1.

### 2.3. Antagonistic Activity of In Vitro Aspergillus terreus Isolate C against Fungal Phytopathogens 

**Figure 3:**
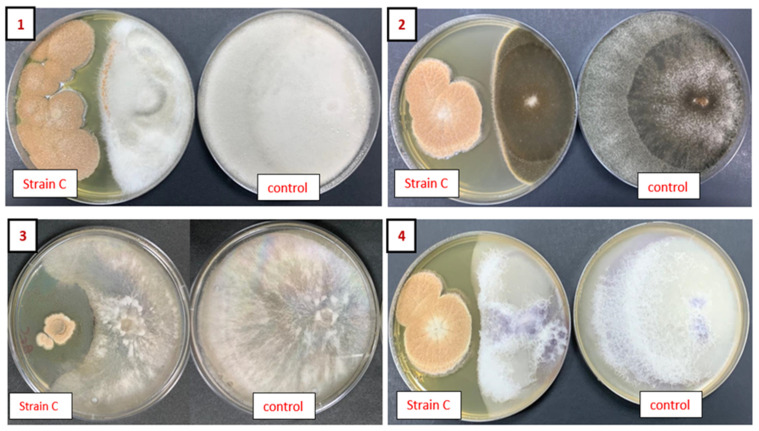
Antifungal activity of endophytic isolate C *Aspergillus terreus* assessed by dual-culture assay against phytopathogens. (**1**) *Colletotrichum gloeosporioides*, (**2**) *Alternaria alternata*, (**3**) *Botrytis cinerea,* and (**4**) *Fusarium oxysporum*.

The antifungal effectiveness of isolate C was assessed using the dual-culture method against four phytopathogens: *Alternaria alternata*, *Botrytis cinerea*, *Fusarium oxysporum*, and *Colletotrichum gloeosporioides*, as shown in Figure 3. The antifungal activity of *A. terreus* strain C was effective against all the phytopathogens used in this study (Table 2). All results were compared with the corresponding phytopathogens grown in control plates.

### 2.4. Screening for the Major Secondary Metabolites of Dichloromethane Extract of A. terreus Analyzed by GC-MS

**Figure 4:**
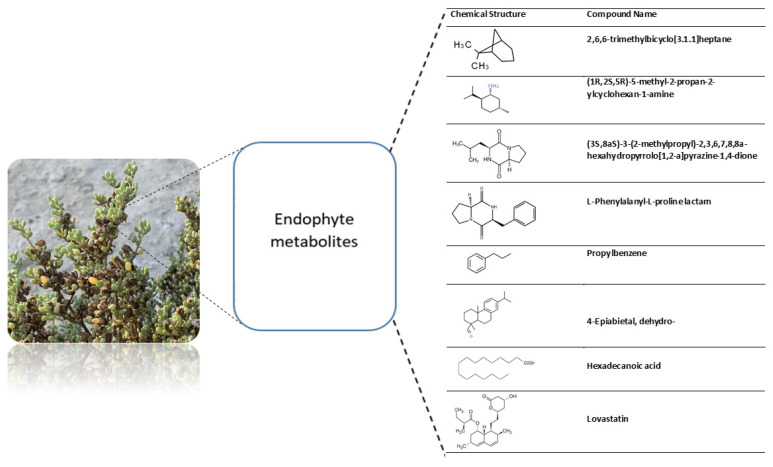
Endophytes and their metabolites.

Fungi utilize secondary metabolites to facilitate their growth and fortify defenses against pathogens. Fungal endophytes, in particular, play a crucial role in supporting plants either directly or indirectly by fostering growth and providing biocontrol against phytopathogens, as illustrated in Figure 4. The identification of these metabolites is instrumental in comprehending their functional roles and discovering compounds with potential benefits.

**Figure 5:**
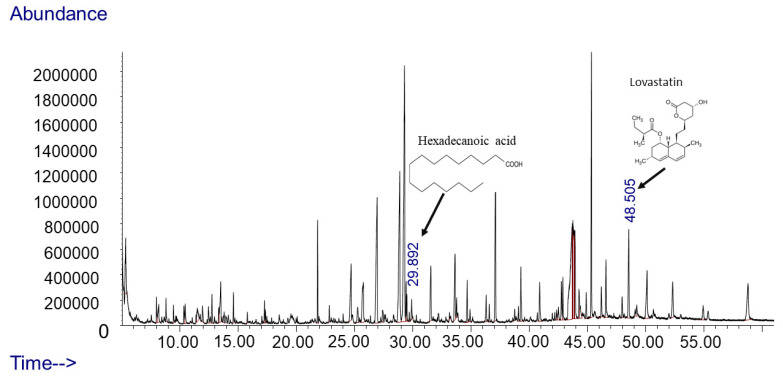
GC-MS scan mood chromatogram of dichloromethane extract of *Aspergillus terreus*.

In the specific case of *A. terreus*, the analysis of its dichloromethane extract using GC-MS revealed the presence of approximately 66 compounds, as depicted in Figure 5. This insight adds to our knowledge of the diverse chemical components produced by fungi, opening avenues for further exploration and application in various beneficial contexts. As an example of these metabolites, *A. terreus* produces hexadecanoic acid and lovastatin, as shown in Figure 5.

### 2.5. The Biocontrol and Growth-Promoting Effects of the Endophytic Isolate Strain C (Aspergillus terreus) on Tomato Seedlings 

Four treatments were considered, namely (T1) untreated seedlings, (T2) seedlings inoculated with fungal endophyte (strain C), (T3) seedlings inoculated with the pathogen *Fusarium oxysporum* spore suspension, and (T4) seedlings inoculated first with fungal endophyte (strain C) and then inoculated with the plant pathogen *F. oxysporum* spore suspension.

**Figure 6:**
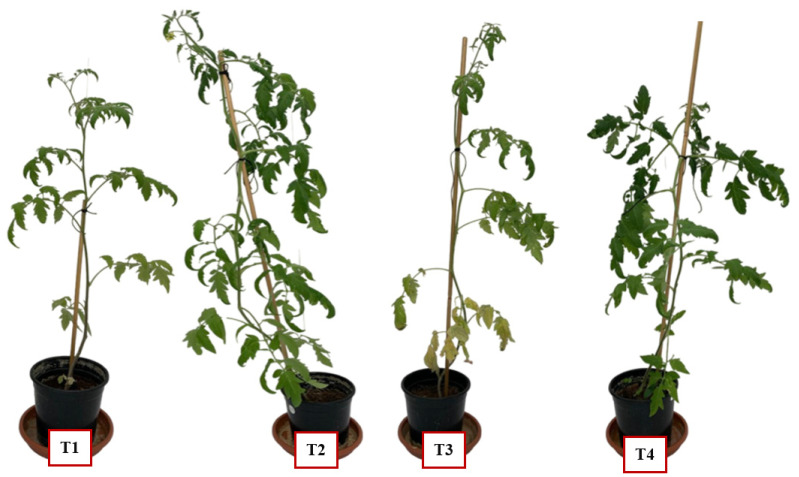
A photo of tomato seedlings after 8 weeks of treatment. (T1) Untreated control, (T2) seedlings inoculated with endophyte strain C, (T3) seedlings inoculated with the pathogen *Fusarium oxysporum,* (T4) seedlings inoculated first (one week earlier) with endophyte strain C and then inoculated with the plant pathogen *F. oxysporum*.

The isolated fungal endophyte (*A. terreus*) was evaluated for its capacity to promote the growth of tomato seedlings under the stress of the fungal pathogen *F. oxysporum*, as shown in Figure 6. The experiment involved four treatments on tomato plants, with growth parameters recorded and presented graphically, including shoot height, chlorophyll content, as well as fresh and dry biomass weights for both above- and below-ground parts.

**Figure 7:**
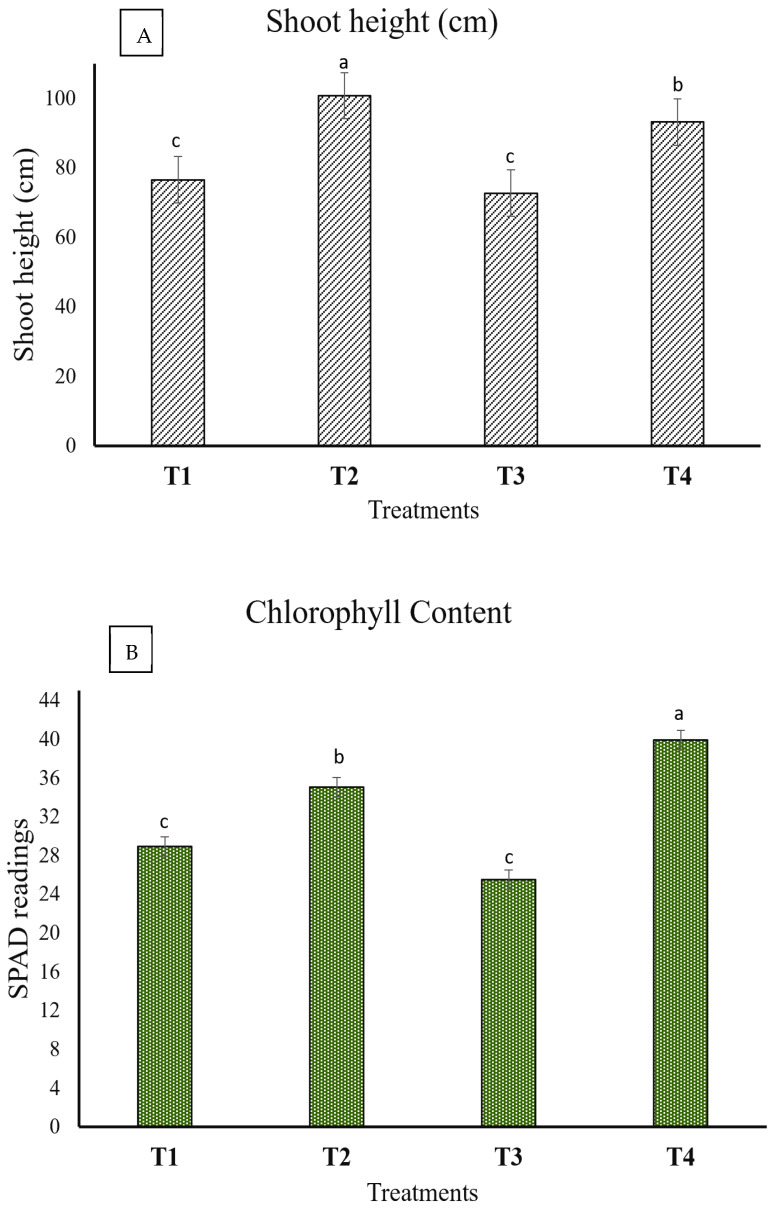
The impact of endophyte and pathogen treatments on the shoot length and chlorophyll content of tomato seedlings was assessed after an 8-week treatment period (N = 4). Graph (**A**) represents the shoot length in (cm). Graph (**B**) represents the chlorophyll content in the SPAD unit. The error bars depict the standard errors of the means. Shared letter(s) among values indicate no significance at *p* ≤ 0.05, as determined by Tukey’s test (N = 4). (T1) Untreated control, (T2) seedlings inoculated with endophyte, *A. terreus* strain C, (T3) seedlings inoculated with the pathogen *Fusarium oxysporum,* (T4) seedlings inoculated first (one week earlier) with endophyte strain C and then inoculated with the plant pathogen *F. oxysporum*.

The overall shoot length was significantly higher in (T2) treatment plants compared to other treatments, as shown in Figure 7A. T2 treatment plants exhibited a shoot height of 100 cm, followed by T4 treatment plants, reaching up to 93.2 cm in shoot length. Conversely, T1 and T3 showed no significant differences in shoot height, with an average length of about 75 cm. The endophytic inoculation promoted seedling shoot length in the absence of the pathogen (T2) and the presence of the pathogen (T4), compared to non-inoculated seedlings (T1) and seedlings inoculated with the pathogen alone (T3), as shown in Figure 7A. In terms of chlorophyll content, the results reflected the pattern observed in shoot length, as shown in Figure 7B. T2 treatment plants displayed the highest chlorophyll content among the treatments.

**Figure 8:**
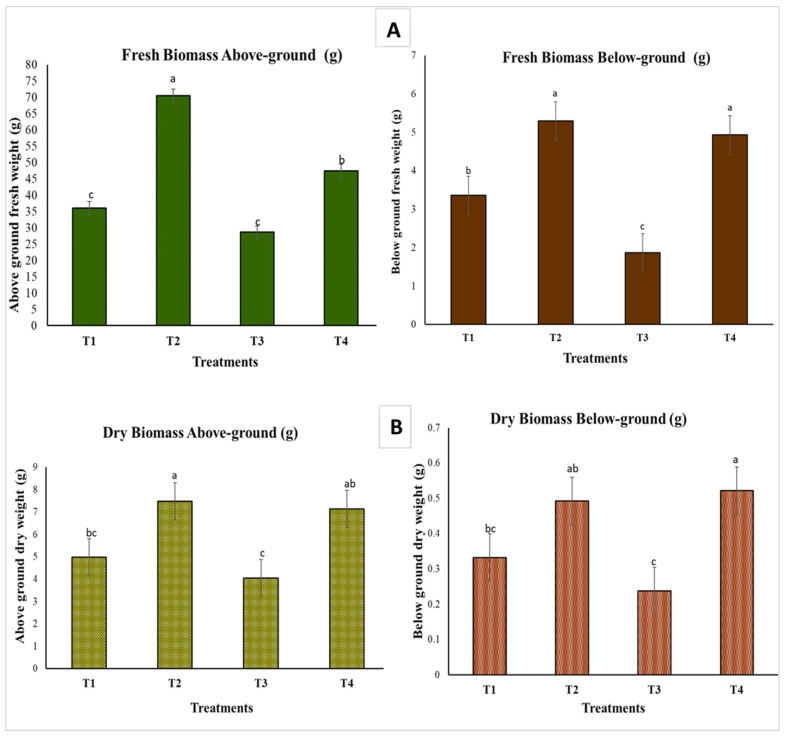
Effect of endophyte and pathogen treatments on the biomass of below-ground and aboveground biomass of tomato seedlings after 8 weeks of treatment (N = 4). (**A**) represents the fresh weight biomass both above and below ground. (**B**) represents the dry weight biomass both above and below ground. The error bars depict the standard errors of the means. Shared letter(s) among values indicate no significance at *p* ≤ 0.05, as determined by Tukey’s test (N = 4). (T1) Untreated control, (T2) seedlings inoculated with endophyte, *A. terreus* strain C, (T3) seedlings inoculated with the pathogen *Fusarium oxysporum,* (T4) seedlings inoculated first (one week earlier) with endophyte strain C and then inoculated with the plant pathogen *F. oxysporum*.

The patterns of fresh and dry biomass for both above- and below-ground parts align with those observed in shoot length and chlorophyll content, as shown in Figure 8. Among the treatments, the seedlings inoculated with the fungal endophyte (*A. terreus*) in treatment 2 (T2) exhibited the highest weights for all recorded values, including both fresh and dry biomass both above and below ground.

### 2.6. Fourier Transform Infrared Spectroscopy (FTIR) Spectral Data Interpretation

**Figure 9:**
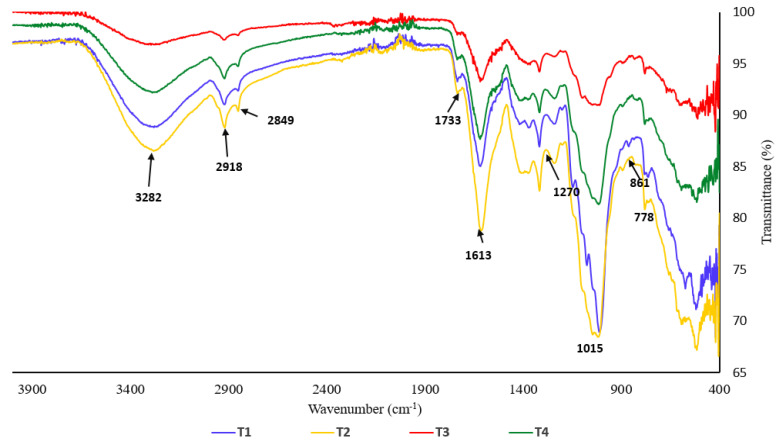
FTIR spectrum of dried leaf powder of tomato under different treatments. (T1) Untreated control, (T2) seedlings inoculated with endophyte, *A. terreus* strain C, (T3) seedlings inoculated with the pathogen *Fusarium oxysporum,* (T4) seedlings inoculated first (one week earlier) with endophyte strain C and then inoculated with the plant pathogen *F. oxysporum*.

Plant tissues consist of numerous substances, leading to intricate spectra with multiple vibrational bands. Compounds responsible for specific characteristics of a spectrum are not easily determined due to their complexity. Nevertheless, it is feasible to identify major classes of compounds present in the samples. In Figure 9, spectra referred to leaves under different treatments (T1, T2, T3, and T4). After the disease was treated with endophyte (T4), the C–H asymmetric vibration bond associated with the characteristic methyl group, usually detected in the range of 2870–2960 cm^−1^, and the bond at 772 cm^−1^ both exhibited a reduction. Additionally, the peak at 1220 cm^−1^, demonstrating the C-N bonding vibration, was detected in the control (T1) and endophyte (T2) cases, but vanished in disease (T3) cases, and the peaks within the range of 1370–1462 cm^−1^ indicate the presence of C=C and C=N bonds. Furthermore, the endophyte (T2) and control (T1) groups had a stronger peak at 1024 cm^−1^, but the disease (T3) group showed a smaller peak. Remarkably, for the specimens subjected to endophytic treatment (T2), certain novel peaks were recognized at 514–593 cm^−1^, 1100–1160 cm^−1^, and 3210–3260 cm^−1^ (O-H and N-H).

## 3. Discussion

Halophytes, a distinct plant group, possess unique traits enabling them to thrive in hostile saline environments with high salt concentrations [27]. Evolution has equipped organisms with various strategies to survive in extreme saline conditions, including inland saline zones, coastal areas, salt marshes, lakes, and mangroves [27,28]. Microbes that exhibit salt tolerance in epiphytic, endophytic, and rhizospheric communities are crucial to the evolution of halophytes in extremely salinated environments [29]. Residing within plants, endophytes play a crucial role in enhancing the plants’ adaptation to their specific ecological environments. By forming a symbiotic relationship with halophytic plants, endophytic fungi in saline environments can enhance salt tolerance. Symbioses improve the plants’ physiological reactions to stress and optimize their defense mechanisms against water salinity [20]. Identifying endophytic fungi that interact with halophytic hosts can provide an avenue for research. These endophytes provide defense against plant pathogens and generate advantageous metabolites that aid host plants in dealing with salt stress [30]. Moreover, they boost the host plant’s growth and development by aiding in nutrient absorption and producing phytohormone siderophore and ACC-deaminase [31]. Additionally, endophytes are renowned for their prolific production of numerous secondary metabolites, many of which hold substantial medicinal value [32].

The presence of sodium chloride ions in soil, leading to soil salinization, hampers plant functions and results in reduced crop yields. Researchers have explored salt-tolerant microorganisms as a strategy to improve crop growth in saline conditions. Our findings indicate that *Aspergillus terreus* (strain C) exhibits tolerance to salinity levels of up to 10%. Our findings align with Wang, Zheng [33], who emphasize the metabolite production of *A. terreus* under 10% NaCl salinity tolerance. This resilience could potentially enhance the host plants’ ability to thrive in challenging environmental conditions characterized by elevated soil salinity.

These fungi possess the ability to produce a broad spectrum of biologically active compounds that demonstrate diverse effects, including insecticidal, antiviral, antioxidant, anticancer, antidiabetic, antifungal, and antibacterial properties, spanning across various structural classes [34,35]. A multitude of natural medicines, including cyclosporine, cephalosporins, lovastatin, rapamycin, and paclitaxel, continue to be employed for immune system suppression and the treatment of conditions such as hypertension, lipid disorders, cancer, and parasitic illnesses. *Aspergillus terreus* is recognized for generating lovastatin, a cholesterol-lowering drug. Additionally, *A. terreus* produces various biologically active compounds, including asterriquinones, butyrolactones, and terretonins [36]. Hexadecanoic acid, also known as palmitic acid, plays a crucial role in controlling phytopathogens and promoting plant growth [37]. It has been proven to inhibit the growth of soil-borne pathogens like *Fusarium oxysporum*, thus aiding in the control of phytopathogens [38]. Studies indicate that palmitic acid can enhance the growth of crop plants and mitigate soil-borne diseases, such as Fusarium wilt [39]. Research emphasizes the antifungal properties of fatty acids, particularly hexadecanoic acid, against phytopathogenic fungi, contributing to effective disease control in plants. In our scan for functional groups, we obtained many compounds, up to 66, indicating further investigation for potential benefits. 

The selected fungal endophyte’s antimicrobial potential was evaluated against four phytopathogenic fungi—*Alternaria alternata*, *Botrytis cinerea*, *Fusarium oxysporum*, and *Colletotrichum gloeosporioides*—using the dual-culture method. The findings demonstrated that the endophytic fungi, specifically isolate C, identified as *Aspergillus terreus*, exhibited antifungal activity against all the investigated fungal pathogens in this study. These results align with the existing literature, reinforcing the observed effectiveness of the fungal endophyte against the tested phytopathogens. Research demonstrated the generation of an inhibition zone by *A. terreus* against *Neurospora crassa* [40]. Additionally, findings from another study indicated that novel compounds from *A. terreus* could induce inhibition zones against the growth of *Pseudomonas aeruginosa* and *Enterobacter aerogenes* [33]. The likelihood of an inhibition zone being formed arises from the secretion of metabolites by *A. terreus* that disrupt the growth of *Pythium aphanidermatum* [41]. *A. terreus* demonstrated significant antifungal efficacy against *Rhizopus oryzae, Mucor racemosus,* and *Syncephalastrum racemosum* [42]. 

Inoculating tomato seedlings with endophytes during phytopathogenic stress can markedly influence the plant’s length, fresh weight, dry weight, and photosynthetic efficiency. Infecting three-week-old tomato seedlings with *Fusarium oxysporum* can cause leaf yellowing, though not necessarily fatal [43,44]. Fusarium wilt, induced by *F. oxysporum*, is viewed as one of the most damaging diseases affecting tomato plants, with symptoms including foliage yellowing and wilting [45]. As illustrated in Figure 6, treatment 3 (T3), where seedlings were solely inoculated with the pathogen (*F. oxysporum*), exhibited yellowish leaves as a clear indication of *Fusarium* disease symptoms. The introduction of endophytic fungi has the potential to improve the photosynthetic efficiency of tomato plants facing stressful conditions, as illustrated in Figure 7B. This enhancement in photosynthetic efficiency can result in improved plant growth and increased resistance to stress [46,47]. The application of endophytic fungi strain C *Aspergillus terreus* has been proven to enhance the fresh and dry weight of tomato plants compared to control plants without inoculation. This weight gain is associated with enhanced photosynthetic efficiency, increased stress resistance, and enhanced nutrient absorption facilitated by endophytic inoculation [48,49]. Introducing endophytes to tomato seedlings experiencing phytopathogenic stress can positively influence their length, fresh weight, dry weight, and photosynthetic efficiency. In future work, the improved plant growth and stress resistance observed can be recognized to investigate the enhanced photosynthetic efficiency, enhanced nutrient uptake, and increased stomatal conductance facilitated by the endophytic inoculation.

A plant growth regulator (PGR) may enter the human body in several ways, including through food and water contaminated by PGR residues, direct skin contact, or inhalation [50]. Sometimes, the levels of certain PGRs in food exceed the allowed limits [51]. The amount of PGRs found in human blood and urine reflects how much exposure there has been to these substances in the environment [52]. Children might have higher concentrations of PGRs in their bodies because they weigh less and may not excrete these substances as effectively as adults [53]. Researchers believe that people who live near farmland tend to have higher levels of PGRs in their urine, with levels varying between genders and ages [54]. Several studies indicate that plant growth regulators (PGRs) can significantly influence insect and animal physiology. For instance, IAA has been associated with neuronal apoptosis and microencephaly [55], while kinetin exhibits antioxidant and antithrombotic properties [56]. PGRs like IAA, ABA, and GA3 have been shown to affect sexual differentiation, physiological parameters, and immune responses in mice [57]. Moreover, PGRs have been linked to teratogenic effects, alterations in cell membrane integrity, and the development of liver neoplasms in animals [58]. In another study, the researchers demonstrated that natural indole-3-acetic acid (IAA) and synthetic 1-naphthaleneacetic acid (NAA) impact the stability, arrangement, and interactions of lipids in model membranes of both plant and animal systems [59]. The extent of these effects varies with the concentration of the auxins, and NAA has a greater impact on plant membranes compared to IAA. At a concentration of 10^−4^ M, which is toxic to both animals and plants, both auxins cause significant disruption. This disruption alters membrane structure, particularly noticeable with IAA. Thus, the damaging effects of IAA and NAA on membranes, especially at higher concentrations, should be taken into account in studies on the toxicity of these substances [59]. These findings underscore the diverse and potentially intricate effects of PGRs on various biological systems. Understanding the factors that affect the toxicity of PGRs in animals is crucial. By considering the exposure methods, physiological hindrance, tissue spreading, and excretion, we can gain valuable insights into the risks involved [50]. These factors play a pivotal role in determining the extent of the harm that PGRs can cause. Therefore, it is essential to take a closer look at these factors and mitigate any potential risks associated with PGR use in animals. There is a need to study the biochemical and physiological impacts of PGRs on antioxidant defense, immune system enzymes, and lipid peroxidation in tissues like the lung and spleen of rats, for example. The toxicity of *Aspergillus terreus*, especially concerning plant growth regulators, is not directly discernible from the search results provided. The results primarily discuss the toxicity of mycotoxins derived from *Aspergillus* and their effects on food, the environment, and human and animal health. Mycotoxins are fungal metabolites that can be harmful to humans, animals, and plants [60]. However, the search results do not specifically address the toxicity of *A. terreus* in plants, soil, humans, or animals in relation to plant growth regulators. To ascertain information on the toxicity of *A. terreus* in these contexts, further research focusing specifically on the toxicity of *A. terreus* plant growth regulators in plants, soil, humans, and animals would be necessary. This could involve investigating the effects of these regulators on plant growth, soil quality, and the potential risks to human and animal health.

## 4. Materials and Methods

### 4.1. Sample Collection and Endophyte Isolation

Fresh leaves of *Tetraena qatarensis* were harvested from the protected field at Qatar University (25°22′6″ N; 51°29′35″ E). To obtain endophytes, 48 healthy plants were meticulously selected for sampling. Leaves from the middle section of each plant were carefully gathered, properly sealed in labeled plastic bags within an icebox, and transported to the laboratory. They were then stored in a refrigerator at 4 °C. The isolation process was promptly carried out within the initial 24 h after collection. Subsequently, the collected leaves went through a cleansing process using running tap water to eliminate any traces of soil or dust. Following this, a surface sterilization procedure was applied to the leaves to eradicate any microbes, specifically epiphytes, present on the leaf surfaces. 

The surface sterilization procedures were conducted as follows. The leaves were immersed in a solution of 2.5% (NaClO) for 1 min, followed by soaking in 70% alcohol for 5 min. Subsequently, the leaves were washed with sterile dH_2_O several times to remove leftovers from sterilization agents. After that, the leaves were cut into parts. Leaf sections were placed onto PDA media plates and incubated at 25 °C for a period of up to 30 days to isolate fungi. The emerging fungi from the inner part of the leaves were sub-cultured to obtain purified isolates. The purified isolates were stored in the refrigerator at 4 °C for later analysis. To assess the effectiveness of surface sterilization, an aliquot of sterile distilled water obtained from the last wash was plated on PDA media and incubated under identical conditions. The absence of microbial growth indicates complete sterilization of the samples. The frequency of colonization for the pieces was calculated by counting the ones which were colonized by the fungal isolates and dividing them by the total number of the applied pieces.

Percentage of colonization = (number of leaves pieces colonized/total number of leaves pieces) ∗ 100%

### 4.2. Identification of Fungal Isolates by Molecular Ribotyping

Extraction of genomic DNA from fresh mycelia (8 days old) was carried out using DNeasy PowerSoil Kit (Qiagen GmbH, Hilden, Germany), as instructed by the company protocol. To identify the isolates, the (ITS) region of the nuclear ribosomal RNA was sequenced using the universal primer pair (ITS1 (forward): 5′-TCCGTTGGTGAACCAGCGG-3′ and ITS4 (reverse) 5′-TCCTCCGCTTATTGATATGC-3′). The Sanger method was used for the sequencing of PCR products. Sequencing results were read using BioEdit software https://bioedit.software.informer.com/. By comparing the amplified sequences of endophytic fungi with available sequences in the National Centre for Biotechnology Information (NCBI) database, Basic Local Alignment Search Tool (BLAST) matching routines were used to identify endophytic fungi. The phylogenetic tree, based on the maximum likelihood method, was constructed using software (MEGA version 11). The current study is part of our broader investigation, where we isolated over 40 strains and grouped them based on morphological characteristics. Among these pure isolates, we genetically identified 21 strains. We specifically chose the *A. terreus* (strain C: ON117337.1) due to its high antagonistic potential against phytopathogens and its abundance (approximately 40%) in the collected samples. The obtained sequence from this study has been deposited in GenBank under the accession number (strain C: ON117337.1). 

### 4.3. Evaluating Salinity Tolerance of Fungal Endophytes

To assess the salinity tolerance of *Aspergillus terreus* isolate, (PDA) medium was used, incorporating varying percentages of NaCl (0%, 2%, 4%, 6%, 8%, 10%). The fungi were then incubated at 25 °C for 10 days, during which daily monitoring of their growth in the presence of NaCl was conducted. This test was conducted in four replicates for each NaCl concentration.

### 4.4. Antagonistic Effect of Aspergillus terreus Isolate against Plant Fungal Pathogens

The dual-culture assay [61] was applied to assess the antagonistic interactions of endophyte strain (C) against the following plant pathogens: *Fusarium oxysporum, Alternaria alternata, Colletotrichum gloeosporioides,* and *Botrytis cinerea.* In this test, a 4 mm diameter disk from the margin of one-week-old fresh mycelium growth was bunched and placed 2 cm away from the margin of the 90 mm PDA plate. The experimental plate containing the phytopathogen and the *A. terreus* isolate were placed on opposite edges, while the control plate containing the phytopathogen disc was placed only 2 cm from the plate border. The PDA plates were incubated at 24 ± 2 °C for 10 days. Four replicates of each treatment were applied, and the results are presented as means ± standard deviation (S.D.). The percentage of pathogenic growth inhibition was calculated according to the following equation: percentage Inhibition = [(C − T)/C] × 100, where T is the radius of the fungal mycelia treated with the endophyte and C is the radius of the fungal mycelia in the control plate.

### 4.5. Secondary Metabolites Extraction and GC-MS Analysis

The extraction of secondary metabolites (SMs) from fungi was performed by preparing the filtrate from the fungal culture broth [62] followed by secondary metabolite extraction according to the EPA 3510C method with modification, briefly described as follows. Fungi were inoculated into a 250 mL flask containing 150 mL purified broth (PD) media and incubated in a shaker incubator at 25 ± 2 °C, at 130 rpm for 2 weeks. Following the incubation, the media were filtered first by centrifuging the culture broth and collecting the supernatant. The supernatant was further filtered by syringe with a 0.45 µm pore size syringe filter. The filtered media were transferred to a 500 mL separatory funnel and acidified by adding 3 drops of concentrated HCl per 100 mL of filtered media. In a fume hood, 15 mL of dichloromethane was added to the acidified media and mixed well. The previous step was implemented three times. The organic solution was moved to a clean bottle and anhydrous sodium sulfate was added (5 gm of anhydrous sodium sulfate for 30 min to take out any excess water). The organic solution was transferred to an evaporation flask for vacuum evaporation using a rotary evaporator. Samples then underwent liquid-to-liquid extraction, followed by concentration to 1 mL using a nitrogen evaporator [63]. Then the sample was collected into an injection vial and analyzed by GC/MS [64]. 

Secondary metabolites were identified through the use of an Agilent Technologies GC system 7890A coupled with a 5973 network mass-selective detector (Agilent Technologies, Inc., Santa Clara, CA, USA). This methodology was adopted from the modified method of Aljabri, Das [65]. The injector temperature was regularly kept at 300 °C. An autosampler was employed to introduce a 1 mL sample (in splitless mode) onto the GC column (30 m × 250 mm × 0.25 mm Rxi 5sil MS column). Helium worked as the carrier gas with a flow rate of 1.67 mL/min and a pressure of 15 psi. The oven temperature was primarily held at 60 °C for 2 min, followed by a rise at a rate of 6 °C/min until it reached 300 °C, where it was kept for 20 min. Various mixtures within the gas stream were ionized using an ion source. The mass spectrometer worked in MS-Scan mode, and the identification of different compounds in the crude samples was achieved using the NIST98 mass spectral database [65].

### 4.6. Greenhouse Experiment

Two-week-old tomato seedlings were propagated in 10 cm diameter pots. Then, the plantlets were inoculated with the fungal endophyte (strain C). The growth was conducted under greenhouse conditions. The study consisted of a single variable with four treatment levels and four replications. The treatments were as follows:

(T1) untreated seedlings;
(T2) seedlings inoculated with fungal endophyte (strain C);
(T3) seedlings inoculated with the pathogen *Fusarium oxysporum* spore suspension;
(T4) seedlings inoculated first with fungal endophyte (strain C) and then inoculated with the plant pathogen *F. oxysporum* spore suspension.

Two-week-old tomato seedlings were subjected to the assigned treatments. For treatments T2 and T4, a fungal suspension (1 × 10^8^ conidial) of strain C *(Aspergillus terreus)* was introduced into the soil, adhering to the roots of the tomato seedlings. This was achieved by soil drenching, which involved applying a fungal suspension to the soil around the roots. Concurrently, for treatment T3, a conidial suspension (1 × 10^8^ conidial) of the pathogenic fungus *F. oxysporum* was inoculated into the seedlings. In the case of T4, the inoculation of the pathogenic fungi *F. oxysporum* occurred one week after the endophytic inoculation.

The plant growth was observed daily for up to 8 weeks. At the end of the experiment, the following growth parameters were measured: (1) leaf chlorophyll contents (using SPAD 502 Plus Chlorophyll Meter), (2) plant heights (measured at harvesting time), (3) fresh weight biomass both above and below ground, (4) dry weight biomass both above and below ground. A Fourier transform infrared spectroscopy (FTIR) technique was used to examine the functional groups (SHIMADZU-IRSpirit, Germany) within the range of 400 cm^−1^ to 4000 cm^−1^. The fine powder of the dried leaves was applied to the diamond crystal of the ATR device.

## 5. Conclusions

In conclusion, the fungal endophytic isolate (*Aspergillus terreus*) emerges as a potential candidate for advancing sustainable agricultural practices. While the current investigation demonstrated a notable enhancement in tomato growth, overcoming the limitation posed by the small number of replicate plants (N = 4) is crucial to ensure the repeatability and robustness of the findings, and additional larger-scale studies are necessary to fully explore the potential of these endophytes. Future research endeavors could focus on elucidating the specific mechanisms through which *A. terreus* contributes to plant health and stress resilience. Investigating its interactions with different plant species and exploring potential applications in diverse agroecosystems would further enhance our understanding. Additionally, assessing the long-term effects of *A. terreus* inoculation on soil health and ecosystem dynamics could provide valuable insights for its practical implementation in sustainable agriculture. Overall, continued exploration of the capabilities of this fungal endophyte holds the potential to revolutionize agricultural strategies for increased resilience and productivity.

## Figures and Tables

**Table 1:** The mycelium growth diameter for strain C on PDA plates mixed with different salinity gradients. The results are the means of four replicates ± S.D.

Salinity Concentration (NaCl)	Mycelium Colony Diameter (mm)
0%	83.58 ± 1.78
2%	80.68 ± 0.82
4%	76.72 ± 1.29
6%	71.17 ± 1.76
8%	68.62 ± 1.11
10%	64.30 ± 0.66

**Table 2:** Effect of *Aspergillus terreus* isolate C on the phytopathogen growth. The results are the means of four replicates ± S.D.

*Pathogenic fungi*	Percentage of Mycelial Growth Inhibition
*Alternaria alternata*	65.9 ± 1.03
*Botrytis cinerea*	73.6 ± 1.08
*Fusarium oxysporum*	63.2 ± 0.96
*Colletotrichum gloeosporioides*	60.2 ± 1.26

## Data Availability

Data are contained within the article.
